# Application of Raman Spectroscopy to Working Gas Sensors: From In situ to *Operando* Studies

**DOI:** 10.3390/s19235075

**Published:** 2019-11-20

**Authors:** Ann-Kathrin Elger, Christian Hess

**Affiliations:** Eduard-Zintl-Institut für Anorganische und Physikalische Chemie, Technische Universität Darmstadt, Alarich-Weiss-Str. 8, 64287 Darmstadt, Germany

**Keywords:** Raman spectroscopy, gas sensors, metal-oxide (MOX) gas sensors, *operando* spectroscopy, In situ spectroscopy, mechanism

## Abstract

Understanding the mode of operation of gas sensors is of great scientific and economic interest. A knowledge-based approach requires the development and application of spectroscopic tools to monitor the relevant surface and bulk processes under working conditions (*operando* approach). In this review we trace the development of vibrational Raman spectroscopy applied to metal-oxide gas sensors, starting from initial applications to very recent *operando* spectroscopic approaches. We highlight the potential of Raman spectroscopy for molecular-level characterization of metal-oxide gas sensors to reveal important mechanistic information, as well as its versatility regarding the design of in situ/*operando* cells and the combination with other techniques. We conclude with an outlook on potential future developments.

## 1. Introduction

Resistive gas sensors, based on semiconducting metal oxides, have been widely used for the detection of gases owing to their high sensitivity and simple fabrication [1,2,3]. These sensors are also referred to as metal-oxide (MOX) gas sensors, chemiresistors or oxidic semiconductor gas sensors. Their basic principle relies on the reversible change in resistance upon exposure to target gases caused by gas adsorption. In the literature, the functions of metal-oxide gas sensors has been described by two models: In the ‘ionosorption model’, the ionosorption of the adsorbates is assumed, transferring electrons from, or to, the sensor’s conduction band. Alternatively, in the ‘oxygen-vacancy model’, the sensor behavior is explained by reduction and reoxidation of the (sub)surface, producing and eliminating oxygen vacancies. The vacancies can be ionized, thereby releasing electrons to the conduction band. In both models, the presence of oxygen (from air) plays an important role, either, as an ionosorbed species or as an oxidation source, but a detailed understanding of the interplay of (sub)surface processes and the sensor signal is still a tremendous challenge [4,5,6]. Part of the complexity of the gas sensor mechanism arises from the fact that, besides the interaction of the target gas with the metal oxide, the analyzed gas may undergo catalytic reactions. Thus, the gas sensing function is not only related to semiconducting, but also the catalytic properties of the sensor material.

For a knowledge-based design of better gas sensors, a detailed understanding of their mode of operation on a molecular level will be required. To identify the gas sensing mechanism, *operando* experiments based on IR, Raman, UV/Vis, and X-ray absorption spectroscopy have been applied [5,6,7,8,9,10,11,12], to relate the sensor response to the structural changes under working conditions. To further enhance the mechanistic understanding of gas sensors, it would be highly desirable to develop *operando* approaches, which (i) are applicable under realistic operating conditions of the gas sensor, (ii) allow for a direct (real-time) correlation of the sensor response with the spectroscopic signals, and (iii) enable a simultaneous monitoring of the gas-phase composition [4,5,6].

An interesting technique regarding the molecular-level characterization of gas sensor materials is vibrational Raman spectroscopy, which can generally be employed without significant interference from gas phase contributions, e.g., strong IR absorbers such as CO_2_ and H_2_O even at elevated temperatures (≥500 °C). Modern Raman spectroscopy on solids is largely applied in a 180° backscattering geometry and by using glass/quartz windows for in situ/*operando* cells, allowing for a versatile cell design. Depending on instrumentation, Raman spectra can cover large sections of the phonon range and can provide useful information on adsorbed oxygen species, which are of particular interest in the context of metal-oxide gas sensors. Major limitations of Raman spectroscopy arise in the presence of fluorescence, which typically lead to the appearance of broad bands dominating the spectrum caused by, e.g., hydroxyl groups, organic impurities or transition metal ions, but may be overcome by changing the excitation wavelength. While, Raman spectroscopy has an inherently low sensitivity in comparison with other optical techniques, the use of modern Raman instrumentation, based on single-stage spectrometers, allows for high throughput and may provide stronger signals.

Despite the potential of Raman spectroscopy for studying gas sensors, only a small number of studies have been published, compared with other characterization techniques (e.g., IR spectroscopy). Previous work published in the context of metal-oxide gas sensors ranges from the (ex situ) use of Raman characterization [13,14,15,16,17,18,19,20] to In situ Raman studies [21,22,23,24,25,26,27,28,29,30,31,32] and *operando* Raman studies [5,6,33,34,35,36], part of which were done in combination with *operando* UV-Vis spectroscopy [6,35]. In these studies typical gas sensor materials, such as SnO_2_, WO_3_, and In_2_O_3_ were investigated towards a variety of target gases, e.g., H_2_S, CH_4_, H_2_, CO, NH_3_, NO_2_, ethanol (EtOH), and acetaldehyde (acetald.). Previous In situ and *operando* Raman studies on metal-oxide gas sensors are summarized in Table 1.

This review is organized as follows: First, we will discuss different In situ and *operando* cell designs, which have been described in the literature for recording Raman spectra during gas sensor operation (see Section 2). Next, the development of Raman spectroscopy, as applied to working metal-oxide gas sensors, will be traced by starting from initial In situ applications (see Section 3) and then moving to very recent *operando* spectroscopic approaches (see Section 4). Finally, the main features of Raman spectroscopy in the context of metal-oxide gas sensors will be summarized and an outlook on potential future developments will be given. Throughout the review, we will illustrate major developments and findings by examples from the literature. 

## 2. Raman Cells for In situ und *Operando* Spectroscopy on Gas Sensors

In situ or *operando* cells, used for studying gas-sensing mechanisms, need to fulfill several basic requirements. Devices must be able to read-out the sensor response paired with the possibility of controlling the temperature of the sensor material. In addition, electrical connections must not interfere with the simultaneous spectroscopic measurements. Furthermore, devices should allow for controlled gas atmospheres and spectroscopic techniques need to be applicable under operation conditions.

**Figure 1:**
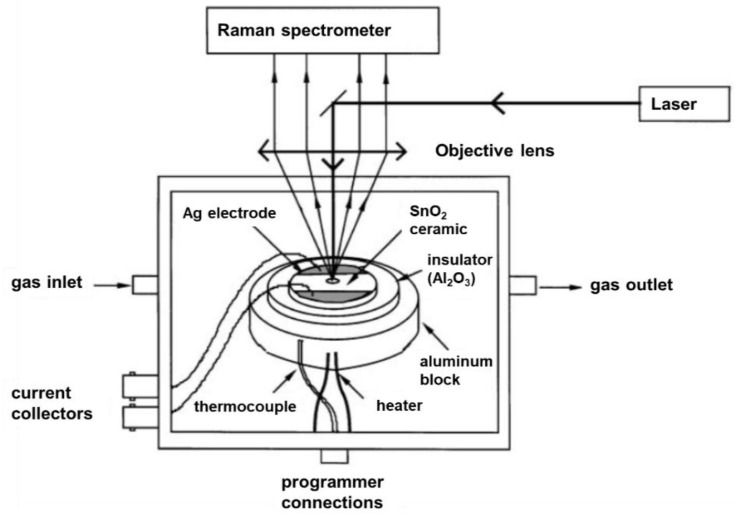
Schematic diagram of an In situ cell used for coupled Raman and impedance spectroscopic measurements. Modified from [22].

**In situ *(operando-pellet) setup***. An early In situ Raman spectroscopic setup in the context of gas sensor applications was reported by Lucazeau and coworkers (see Figure 1) [22,24,28]. The setup allows for simultaneous Raman and impedance spectroscopic measurements at controlled temperatures of the sensor material and at defined gas feed. At the top, the stainless steel cell is equipped with a quartz window enabling visible Raman spectroscopic experiments in 180° backscattering geometry. The sample pellets are placed in an alumina crucible and contacted with different electrode materials depending on the used metal-oxide sensor material (e.g., Ag electrode on SnO_2_ [22], Au on WO_3_ [24], Pt electrode on BaCeO_3_ [28]). Al_2_O_3_ serves as an electrical insulator and the filled crucible is placed on a heating block. The setup enables the direct correlation between changes on the surface of the material, e.g., adsorbates, by Raman spectroscopy and the sensor resistance. However, the gas-phase composition at the outlet of the cell was not monitored. 

**Figure 2:**
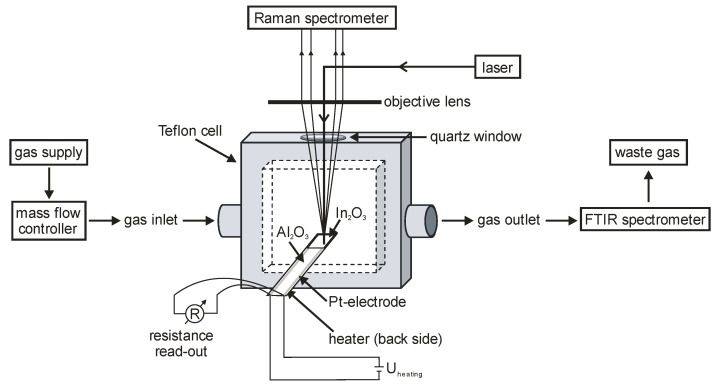
*Operando* Raman spectroscopic setup for simultaneous measurement of the sensor response (dc electrical conductivity), Raman spectra of the sensor material and FT-IR spectra of the gas-phase composition. Reproduced from [5].

***Operando setups***. Figure 2 shows an *operando* Raman spectroscopic setup allowing for coupled Raman spectroscopic and resistance measurements, while simultaneously, the gas-phase composition is monitored by IR spectroscopy [5]. The specifically designed *operando* cell is made of Teflon, equipped with a quartz window for Raman spectroscopic measurements in 180° backscattering geometry, and a gas in- and outlet. The interdigital transducer (Al_2_O_3_, Pt electrode) is coated with the porous sensing layer to enable resistance measurements. The temperature of the sensor is controlled by a Pt heater on the back side of the sensor chip. To minimize condensation, the tube between gas outlet and the IR spectrometer is constantly heated at 100 °C.

To gain further insight into the sensor process, the above described *operando* setup has been extended by UV-Vis spectroscopy to a *multi operando*-approach [6]. Supplemental to Raman and FT-IR measurements, UV-Vis spectra allow monitoring the number of oxygen vacancies in the metal-oxide during the sensor process as discussed below.

## 3. Towards *Operando* Raman Spectroscopy on Gas Sensors 

Despite the potential of Raman spectroscopy for studying gas sensors at work, only a few In situ Raman studies on metal oxide gas sensors have been published [21,22,23,24,25,26,27,28,29,30,31,32], which in part were done with simultaneous recording of the resistance [22,23,24]. For nanocrystalline SnO_2_ [22] and CuO/SnO_2_ [23] at 100 °C, upon exposure to 300 ppm H_2_S, the reversible formation of sulfide species (SnS*_x_*, Cu_2_S) was reported, which was correlated with the simultaneously measured decrease in resistance (*operando*-pellet approach). 

**Figure 3:**
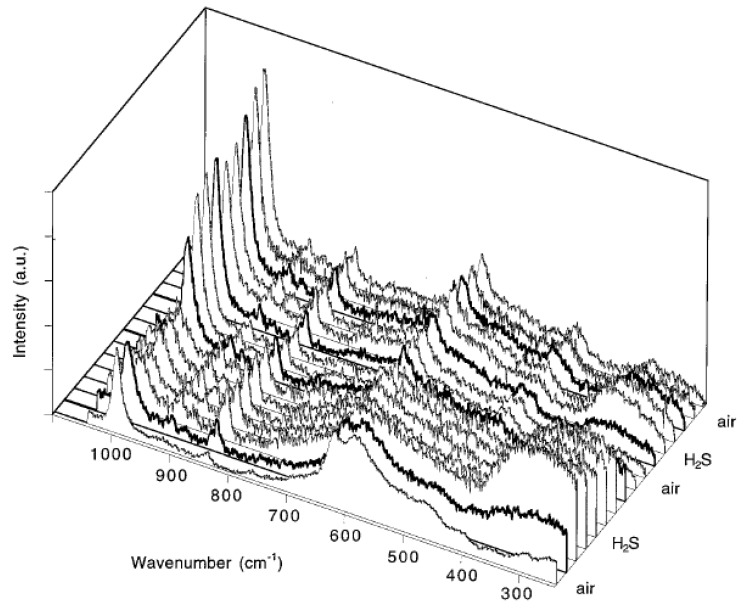
In situ Raman spectra of a SnO_2_ pellet during exposure to two 300 ppm H_2_S-air cycles at 100 °C. Bold lines indicate the first spectra recorded after atmospheric change. Reproduced with permission from [22].

Figure 3 shows the In situ Raman spectra of a SnO_2_ pellet synthesized by hydrolysis of SnCl_4_ [22]. The atmosphere was switched two times between dry air and 300 ppm H_2_S in nitrogen at 100 °C. The initial Raman spectrum in air is dominated by the A_1g_ line of SnO_2_ at 618 cm^−1^, surface modes between 450 and 700 cm^−1^ and a signal at 990 cm^−1^, which is proposed to be caused by the presence of SO_4_^2−^ ions [22]. After switching to H_2_S atmosphere, the whole Raman spectrum intensity decreases and a broad band, at around 350 cm^−1^, appears. The latter is assigned to be a sign for the appearance of superficial SnS_x_. Parallel to the spectroscopic changes Pagnier et al. observed a decrease in the sensor resistance. When switching back to dry air, the resistance and the whole spectrum intensity increase except the surface modes, which stay decreased, and the signal at around 350 cm^−1^, which disappears. The band at 990 cm^−1^ increases due to the presence of oxygen. A second cycle shows a similar behavior as the first, with the exception that the surface modes stay decreased, and thereby, the E_g_ mode of SnO_2_ at 476 cm^−1^ becomes visible. On the basis of the In situ Raman data the drop in resistance after switching to H_2_S is related to the formation of sulfurous (or sulfuric) species. Besides, the authors do not see a connection between the presence of adsorbed sulfate anions and changes in the resistance.

**Figure 4:**
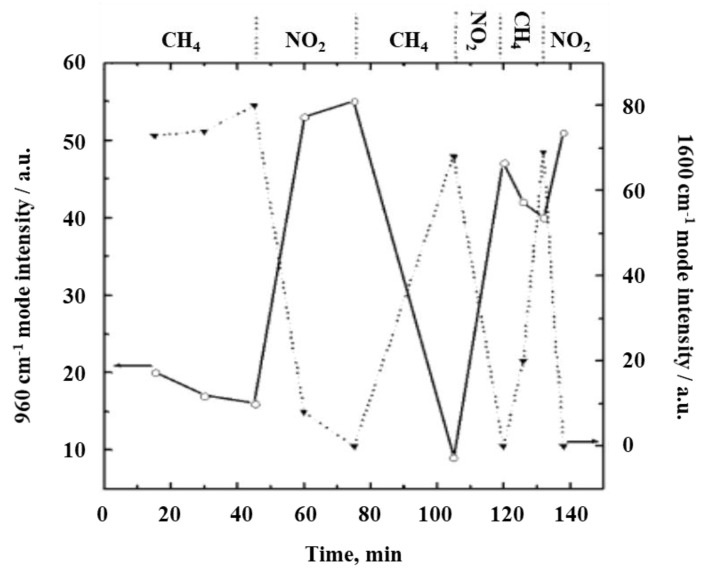
Changes in the Raman intensity of the 960 (solid) and 1600 cm^−1^ (dotted) bands of WO_3_ (2 nm particle size) during varying atmospheres. Modified from [24].

In studies on nanostructured WO_3_ sensors with different grain sizes exposure to 10% CH_4_/H_2_ and 1.8% CO/N_2_ at 150 °C resulted in the formation of carbon species and an increase in conductivity, whereas under oxidative conditions (1000 ppm NO_2_), the carbon species disappeared and the conductivity significantly decreased [24]. Figure 4 depicts the changes in the Raman intensity of the bands at 960 and 1600 cm^−1^. Boulova et al. assign the former to WO_3_ hydrates and the latter to carbon species. Note that second-order bands of carbon species around 2600–3300 cm^−1^ confirm this observation. The coverage of the surface with carbon species during CH_4_ exposure leads to a decrease in the intensity of the 960 cm^−1^ mode, thus, indicating its surface character. The sample with the smallest particle size (2 nm) exhibits the highest sensitivity to the presence of carbonaceous molecules. In contrast, Raman spectra of samples with larger particle size (>35 nm) show no changes during exposure to different gases due to the grain size effect, i.e., with increasing grain size the percentage of surface atoms decreases and the 960 cm^−1^ signal disappears from the Raman spectrum.

In a further study on metal-oxide gas sensors, Ou et al. applied In situ Raman spectroscopy to Pd/WO_3_ thin films exposed to 1% H_2_ in synthetic air at different temperatures (20, 60, 100, and 140 °C) [29]. In the presence of H_2_, new Raman signals appear at 331 cm^−1^ and 656 cm^−1^, which were attributed to *ν*(O–W^5+^–O) and WO_3_·H_2_O *ν*(O–W–O) stretching modes, respectively. When switching the atmosphere to dry air at 20 °C, the signal at 656 cm^−1^ remains almost unaffected, indicating that the formed surface water is stable under these conditions. At temperatures above 100 °C, both signals decrease due to surface water desorption and recombination of oxygen vacancies.

The effects of increasing temperature on the Raman spectral characteristics of different, undoped WO_3_ gas sensors (monoclinic WO_3_ on Si substrate, nanopowder, nanowires) were studied by Garcia-Sanchez et al. [26]. In the temperature range between 30 and 160 °C under environmental conditions the intensity of W-OH-related vibrational modes increases with temperature. This is explained by the stronger reaction of the W-OH bonds to raised temperature than that of the normal W-O bonds of the original lattice structure. Consequently, the operating temperature does not only affect the Raman shift but also the intensity ratios.

**Figure 5:**
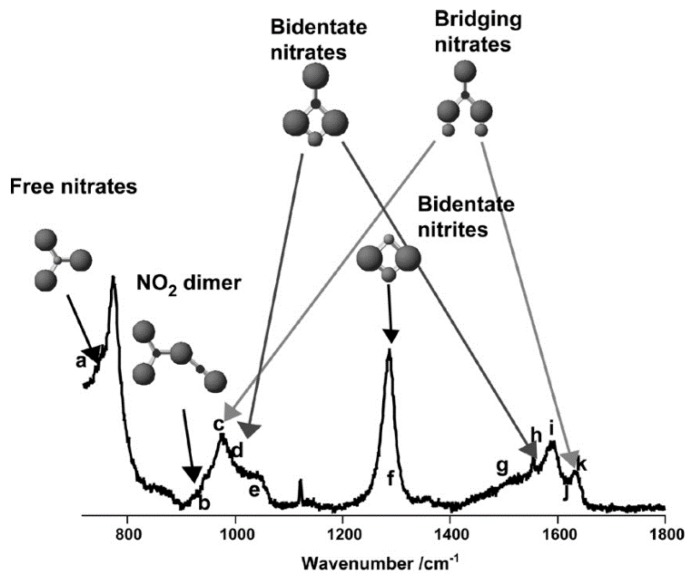
In situ Raman spectrum of SnO_2_ in 1000 ppm NO_2_ atmosphere at 25 °C. The assignment of the peaks is as follows: a,e—free NO_3_; b—NO_2_ dimer; c, j, k—bridging nitrates; d, g, h, i—bidentate nitrates; f—bidentate nitrites. Reproduced with permission from [31].

In the context of gas sensing, the adsorption of 1000 ppm NO_2_ on nanocrystalline SnO_2_ was investigated by Sergent et al. within a temperature range of 25–500 °C using In situ Raman spectroscopy [25,31]. During heating, in NO_2_ atmosphere, several Raman bands are observed and attributed to the formation of NO_2_ dimers, nitrite, bridging, and bidentate nitrate (see Figure 5). Based on the analysis of the temperature-dependent adsorption behavior of the different NO*_x_* species, the conductivity decrease of SnO_2_ in the presence of NO_2_ is associated with the formation of nitrite species. Analogous measurements were performed for TiO_2_ sensors, revealing similar but less conclusive results, owing to the reduced intensity of the adsorbed species [31]. Recently, In situ Raman spectroscopy coupled with simultaneous FT-IR gas-phase analysis has revealed new information about the dynamics of the (sub)surface structure of ceria upon NO*_x_* exposure at 30 °C, such as the participation of Ce-O surface sites, besides the identification of nitrite and nitrate adsorbates [37].

**Figure 6:**
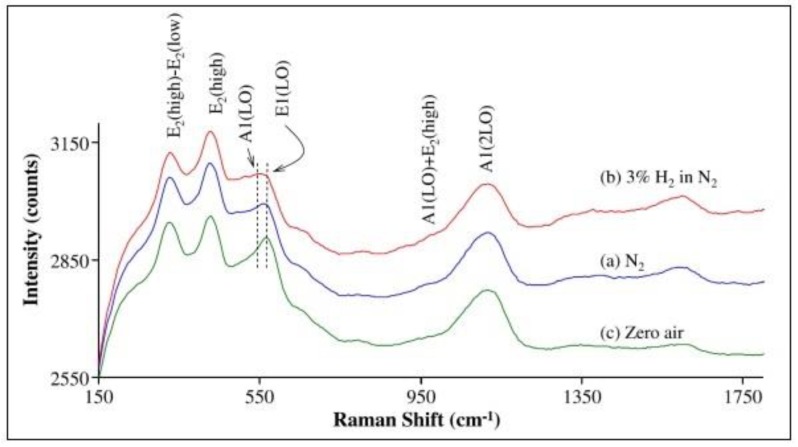
In situ Raman spectra of ZnO (sample A) in (a) N_2_, (b) 3% H_2_ in N_2_, and (c) zero air. The spectra are offset or clarity. Modified from [30].

ZnO, the first metal-oxide used for gas detection [4], was examined by In situ Raman spectroscopy in combination with ex situ XRD and FT-IR experiments to gain insight into the hydroxyl group formation in H_2_ atmosphere [30]. Raman spectra were measured on a ZnO nanopowder (average grain size <50 nm) at 300 °C in different gas atmospheres (N_2_, 3% H_2_/N_2_, zero air). As shown in Figure 6, switching from pure N_2_ to 3% H_2_ in N_2_ leads to altered signals within 400-600 cm^−1^. In fact, the ratio of the A_1_(LO) band at 543 cm^−1^ and the E_1_(LO) band at 566 cm^−1^ increases under reducing conditions, whereby the E_1_(LO) mode has been associated with the formation of surface defects (oxygen vacancies or Zn interstitials). Subsequent exposure to an oxygen-containing atmosphere leads to an intensity increase of the E_1_(LO) feature, while the A_1_(LO) mode shows only minor changes. 

In the presence of H_2_, an increase in the baseline at around 480 cm^−1^ can be observed, which the authors attribute to the formation of surface hydroxyl groups, as assisted by XRD and FT-IR measurements. Using Raman spectroscopy and XRD, they also examined the influence of crystallinity and average grain size on the formation of surface hydroxyl groups on ZnO. As a measure of crystallinity, the Raman band at 435 cm^−1^ (E_2_(high)) can be employed, which originates from the oxygen vibration in the ZnO crystal lattice. For a sample with reduced crystallinity and an average grain size <50 nm (see Figure 6) a lower intensity of the 435 cm^−1^ band is observed, compared to a sample with a higher crystallinity (not shown) and increased average grain size (several hundreds of nanometers), which showed no changes at around 483 cm^−1^. It is worth mentioning that Boulova et al. observed a similar behavior for WO_3_ gas sensors towards CH_4_/H_2_ exposure (see above) [24], indicating the more general importance of the crystallinity and grain size of metal oxides for gas sensing applications.

## 4. Application of *Operando* Raman Spectroscopy to Gas Sensors

In the following, the progress in the application of *operando* Raman spectroscopy to metal-oxide gas sensors will be outlined. Earlier work by Pagnier et al. in 1999 on H_2_S sensing over SnO_2_-based pellets introduced the coupling of In situ Raman spectra with resistance measurements (*operando*-pellet approach, see above) [22,23]. In 2013, the first *operando* Raman spectroscopic study was published by Sänze et al. on EtOH sensing using In_2_O_3_ gas sensors [5]. In this work, the resistance was measured simultaneously with Raman spectra and gas-phase IR spectra to relate the sensor response with the presence of adsorbates, changes in the metal-oxide material, and the gas-phase composition (see Section 4.1). Since then further developments in the *operando* methodology lead to *operando* SERS (see Section 4.2) and combined *operando* Raman/UV-Vis approaches (see Section 4.3), as illustrated in EtOH detection on In_2_O_3_-based, and SnO_2_ gas sensors, respectively [6].

### 4.1. Operando Raman Spectroscopy

**Figure 7:**
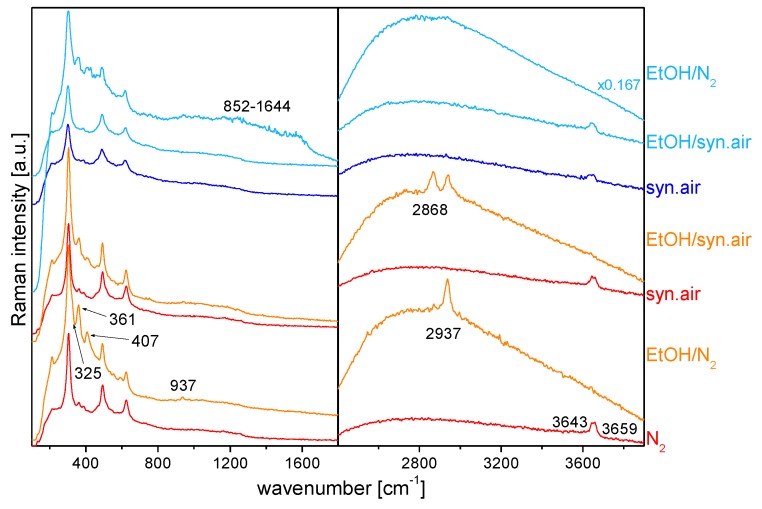
*Operando* Raman spectra (at 514.5 nm excitation) of the ethanol gas sensing of indium oxide at 190 °C (red/orange) and 325 °C (blue). Spectra are offset for clarity. Modified from [5].

***In_2_O_3_ gas sensors.*** In the first example of the application of *operando* Raman spectroscopy, we will discuss the EtOH detection by indium oxide gas sensors [5]. In this study, a simultaneous measurement of the resistance, Raman spectra and gas-phase IR spectra during sensor operation was performed, but here we will focus on the discussion of the Raman spectra. Figure 7 depicts *operando* Raman spectra (514.5 nm) of the In_2_O_3_ gas sensor recorded in different gas environments at 190 °C (red/orange) and 325 °C (blue). Starting at 190 °C in nitrogen, the phonon region is characterized by oxide bands at 304, 361, 493, and 624 cm^−1^, confirming the presence of bixbyite-type c-In_2_O_3_, [5,38]. Whereas, the high-frequency region shows the presence of (bridging) hydroxy bands at 3643 and 3659 cm^−1^.

When the sensor is exposed to 250 ppm EtOH/N_2_, several distinct changes are observed: The intensity of the 361 cm^−1^ band of an In-O-In stretch vibration increases. A new band and a shoulder appear at 407, and 325 cm^−1^, respectively, which originate from reduced indium oxide species near the surface [39]. Upon exposure to oxygen the latter bands disappear indicating that near-surface regions can reversibly be switched between an oxidized and a reduced state. In addition, the presence of ethanol results in the disappearance of the hydroxy band at 3659 cm^−1^ and an intensity decrease of the 3643 cm^−1^ band, while new Raman bands appear at 937 (C-C symmetric stretch) and 2937 cm^−1^ (CH_3_ symmetric stretch) [40]. These bands were also observed during the reaction of acetaldehyde with indium oxide under the same conditions. Thus, the observed spectral changes can be associated with the reaction of ethanol resulting in the formation of acetate groups. When switching to synthetic air, the intensity of the acetate features decreases and those of the hydroxy bands increases again. As shown in Figure 7, subsequent exposure to 250 ppm EtOH/syn. air results in indium oxide reduction, but to a smaller degree, as compared to EtOH/N_2_. In contrast to EtOH/N_2_, the presence of oxygen leads to the appearance of a new strong Raman band at 2868 cm^−1^ (besides the bands at 937 and 2937 cm^−1^), which is assigned to the CH symmetric stretch of a formate-like species [41]. In summary, our findings at 190 °C demonstrate that, depending on the gas environment, the Raman spectrum of the In_2_O_3_ gas sensor is characterized by a specific spectral signature, creating a basis for a correlation of the sensor response with the spectroscopic features.

Turning now to the *operando* Raman spectra at 325 °C (shown in blue), the spectrum in synthetic air resembles the previously described spectrum in synthetic air recorded at 190 °C. Likewise, addition of EtOH (250 ppm EtOH/syn. air) does not induce any significant changes in the Raman spectrum. In contrast, upon exposure to 250 ppm EtOH/N_2_, dramatic spectral changes are observed: First, the intensity of the features at 325 and 407 cm^−1^ increases, which is indicative of indium oxide reduction. Second, new broad bands appear at 852–1644 cm^−1^ and at 2670–2958 cm^−1^, which are attributed to carbon, and CH_x_, respectively, and originate from adsorbate decomposition. The behavior at 325 °C strongly contrasts that at 190 °C. Gas-phase IR spectra (not shown here) show an increased formation of products at higher temperature. Thus, surface processes are much faster at 325 °C and as a consequence, the presence of adsorbate species on the sensor surface cannot be observed anymore.

**Figure 8:**
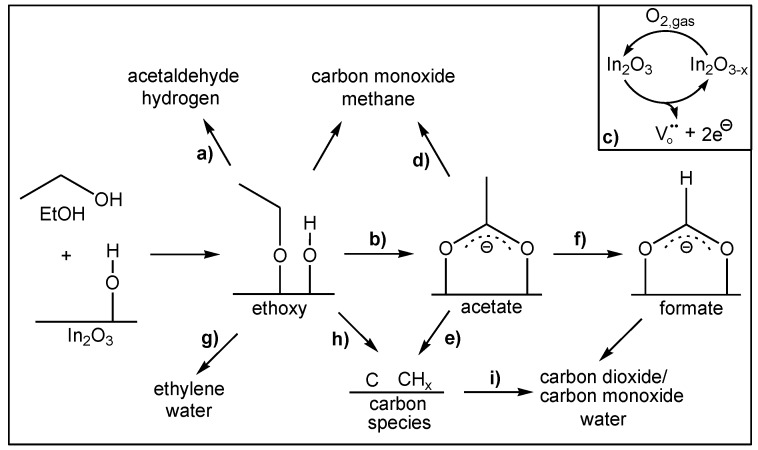
Proposed mechanism of the ethanol gas sensing of indium oxide. For details see text. Reproduced from [5].

On the basis of a large number of experiments, which are detailed in [5,34], a reaction mechanism of EtOH gas sensing on In_2_O_3_ gas sensors was proposed (see Figure 8). In the following, we will summarize its main features. Adsorption of ethanol on the indium oxide surface leads to ethoxy formation, which can either desorb as acetaldehyde; (a) or react with surface hydroxyl groups to form acetate (b). In the course of the redox reaction indium oxide near the surface is reduced (c), releasing electrons in the conduction band thus inducing a decrease in the sensor resistance. A second contribution to the conductivity for ethanol gas sensing is the high stability of the adsorbed acetate. Only thermal decomposition of acetate (d,e) enables the sensor to start returning to its initial surface state, while in the presence of oxygen, acetate partly decomposes to formate-like species (f). 

**Figure 9:**
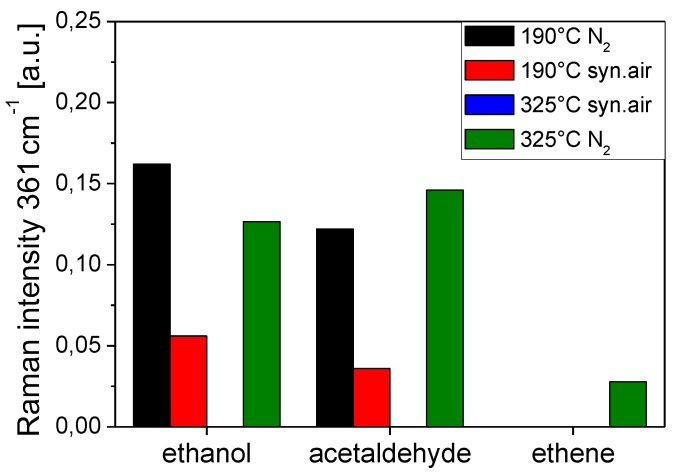
Influence of gas environment and temperature on the degree of reduction of an indium oxide gas sensor. The intensity of the Raman band at 361 cm^−1^ was normalized to that at 307 cm^−1^ and corrected for the intensity in the carrier gas. Reproduced from [32].

In the context of the mechanistic study on EtOH sensing by In_2_O_3_ gas sensors discussed above, experiments with other analytes were performed [34]. Figure 9 compares the influence of the gas environment/temperature on the degree of reduction of the gas sensor for ethanol, acetaldehyde, and ethene. Focusing on the 190°C data, where the (sub)surface processes are sufficiently slow to allow for detailed spectral analysis, the degree of reduction decreases in the order ethanol > acetaldehyde >> ethene; in the presence of oxygen, the degree of reduction was smaller in all cases. The observed behavior can be associated with the different reaction pathways to acetate. As for ethanol, one more hydrogen atom needs to be separated compared to acetaldehyde, the carbon oxidation state needs to undergo a larger change in case of the ethanol reaction.

***CeO_2_-based gas sensors.*** A second example for the application of *operando* Raman spectroscopy, we will discuss the EtOH detection by ceria-based gas sensors [36]. While, the resistance, Raman spectra, and gas-phase IR spectra were measured simultaneously during sensor operation, focus will be put again on the discussion of the Raman spectra. In particular, Raman spectroscopy is shown to allow the direct observation of oxygen vacancies in working gas sensors for the first time, by monitoring the F_2g_ band position [36]. Furthermore, by combining *operando* Raman spectroscopy with theoretical calculations, we can quantify the changes in the oxygen vacancy concentration in the subsurface upon exposure to ethanol.

**Figure 10:**
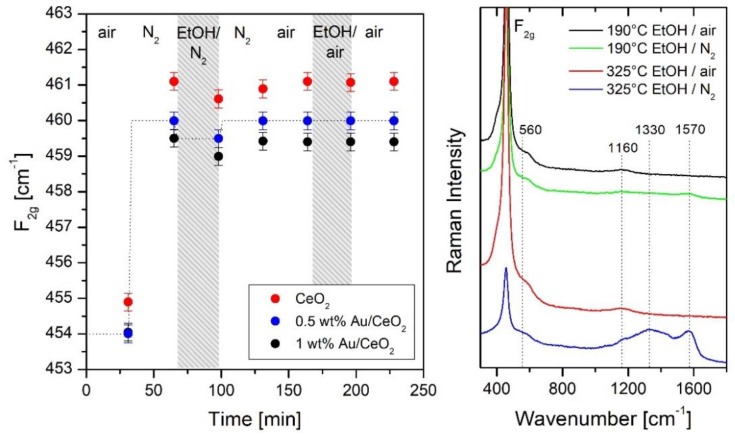
**Left:** Comparison of F_2g_ band positions for CeO_2_, 0.5 wt.% Au/CeO_2_, and 1 wt.% Au/CeO_2_ gas sensors in different gas environments at 190 °C after initial calcination in air at 400 °C. The dashed line is a guide to the eye. **Right:** Raman (514.5 nm) spectra of the ethanol gas sensing by 0.5 wt.% Au/CeO_2_. Spectra are offset for clarity. Modified from [36].

The left of Figure 10 depicts a comparison of the F_2g_ band positions for CeO_2_, 0.5 wt.% Au/CeO_2_, and 1 wt.% Au/CeO_2_ gas sensors during ethanol gas sensing at 190 °C (250 ppm EtOH/N_2_, for details see [34]). Previously, it has been shown based on theoretical studies [36], that there is a linear relationship between the redshift of the F_2g_ band position and the changes δ in ceria stoichiometry CeO_2-x-δ_ due to increasing oxygen-vacancy concentration. Thus, the position of the F_2g_ mode can serve as a quantitative indicator for changes in the oxygen-vacancy concentration, with a redshift of 1 cm^−1^ corresponding to a change in stoichiometry of δ = 0.024. According to Figure 10, Au-loaded samples show a stronger redshift in the initial F_2g_ band position at 400 °C, compared to bare ceria, corresponding to a higher oxygen-vacancy concentration, which is consistent with the prediction from theory and previous experimental studies [42,43]. Upon cooling to 190 °C the F_2g_ band experiences a pronounced blueshift as a result of lattice contraction [36]. 

According to Figure 10, all ceria-based sensors show a comparable redshift of the F_2g_ band in the presence of 250 ppm EtOH/N_2_ despite their different initial state, underlining the importance of the used semiconductor material for oxygen-vacancy formation. If EtOH is switched off, reoxidation of the sensor (subsurface) sets in, as confirmed by the observed F_2g_ blue-shift (see Figure 10). Note that under these conditions the resistance does not show a significant variation, i.e., the resistance does not correlate with the observed decrease in ceria oxygen-vacancy concentration (not shown). This behavior strongly suggests that the detected changes in subsurface oxygen-vacancy concentration are not responsible for the sensor response, but rather, surface oxygen vacancies and adsorbates, as discussed below (see Section 4.3). 

The right of Figure 10 depicts typical Raman spectra of 0.5 wt.% Au/CeO_2_ recorded in different gas environments at 190 °C (black/green) and 325 °C (red/blue). During EtOH gas sensing (250 ppm EtOH/N_2_) at 325 °C, *operando* Raman spectra show broad features at around 1330 and 1570 cm^−1^ due to carbon D and G bands are observed, indicating the decomposition of EtOH on the sensor surface, somewhat resembling the behavior of In_2_O_3_ gas sensors discussed above (see Section 4.1).

### 4.2. Operando Surface-Enhanced Raman Spectroscopy (SERS)

Doping In_2_O_3_ with Ag had a positive effect on the performance of the gas sensor towards EtOH detection. For example, for an In_2_O_3_ gas sensor doped with ~0.5 wt.% Ag at the surface, exposure to 250 ppm EtOH/O_2_ at 190°C, resulted in an increase of the sensitivity and the recovery by 3, and 38, respectively, as compared to bare In_2_O_3_. As illustrated above for In_2_O_3_ gas sensors during EtOH gas sensing, *operando* Raman studies allow the sensor signal to be directly correlated with the nature of the adsorbates, the presence of surface hydroxyl groups and the indium oxide oxidation state. However, in case of doped metal-oxide gas sensors, the presence of a noble metal may decrease the Raman scattering, significantly making a detailed Raman spectroscopic analysis more difficult or sometimes impossible. To overcome these limitations surface-enhanced Raman spectroscopy (SERS) may be employed, which enables the sensitive analysis of surfaces, owing to the enhancement of the Raman signal in the presence of a noble metal. It is generally accepted in the literature that SERS originates from electromagnetic (EM) enhancement and charge transfer (CT) enhancement [44]. EM enhancement is primarily related to the presence of surface plasmons of the noble metal substrate. To this end, Au- and Ag-based SERS substrates have been shown to exhibit large enhancement factors at visible excitation wavelengths. 

In the context of metal-oxide gas sensing materials, SERS effects have previously been observed for Ag und Au doped materials such as Au/SnO_2_ [45] and Ag/In_2_O_3_ [15]. It has been shown that SERS effects may also become operative in case of core-shell systems, e.g., Au@Pd, by which the surface properties are determined by palladium, but the gold core ensures absorption in the visible [46]. Despite its potential, SERS has not been employed to study metal-oxide gas sensors under In situ or *operando* conditions. 

**Figure 11:**
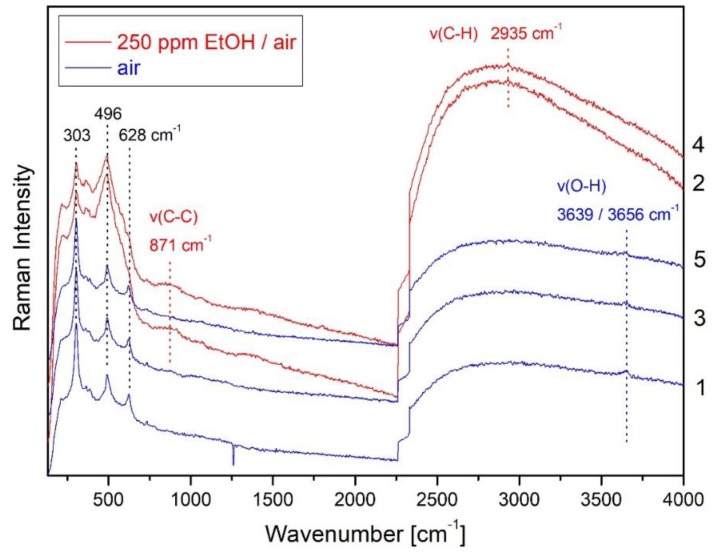
*Operando* surface-enhanced Raman spectroscopy (SERS) during EtOH sensing at 190 °C using In_2_O_3_ doped with Ag. Raman spectra were recorded at 514.5 nm excitation when switching between air (blue) and EtOH/air (red). Spectra are offset for clarity. Modified from [35].

To explore the state of silver origin during working conditions, we have recently exploited the potential of *operando* SERS, for the first time in the context of metal-oxide gas sensors [35]. Figure 11 depicts 514.5 nm Raman spectra for Ag/In_2_O_3_, when switching between air (spectra 1, 3, 5) and 250 ppm EtOH/air (spectra 2, 4) at 190 °C. In air, the gas sensor is characterized by Bixbyit indium oxide features at 303, 363, 496, and 628 cm^−1^ (see Section 4.1) and O-H stretching bands at 3639 and 3656 cm^−1^ of (bridging) hydroxy groups. The disappearance of the O-H bands upon exposure to EtOH/air is accompanied by the appearance of acetate-related bands at 871 cm^−1^ (C-C stretch) and 2935 cm^−1^ (C-H stretch). Please note that exposure to 250 ppm EtOH/air leads to dramatic changes in the low wavenumber region, which are reversible and can be related to the change in the Ag oxidation state during EtOH sensing. XPS analysis of the Ag/In_2_O_3_ gas sensor have revealed that about half of the silver present at the surface is oxidized. In contrast, the strong intensity increase in the In_2_O_3_ phonons in Figure 11 is attributed to the surface reduction of oxidized to metallic Ag giving rise to the observed SERS induced enhancement of the Raman signal. This conversely means that *operando* SERS experiments enable the metal oxidation state of working gas sensors to be elucidated.

### 4.3. Multiple Operando Spectroscopy

**Figure 12:**
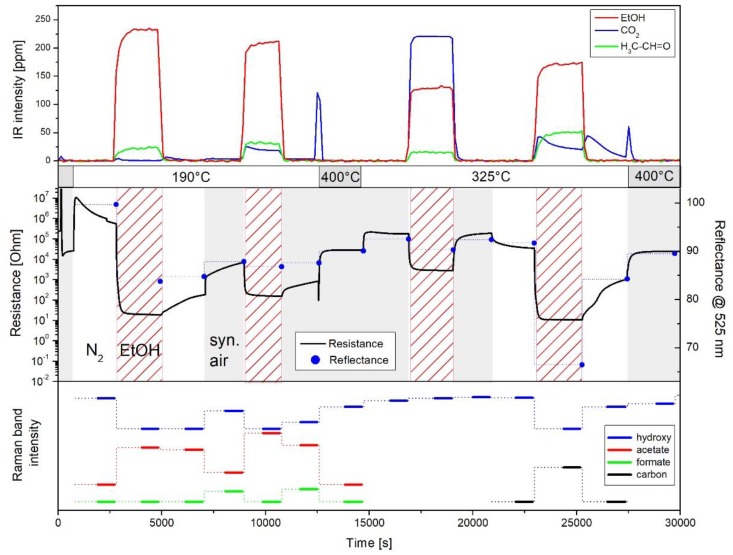
Temporal correlation of spectroscopic data and sensor resistance of the combined *operando* UV-Vis/Raman (514.5 nm)/IR experiment during ethanol gas sensing of SnO_2_. Dashed lines are a guide to the eye. Raman band intensities of hydroxy and acetate species are offset for clarity. Reproduced from [6].

Owing to the complexity of metal oxide gas sensors a combination of techniques in one experimental setup is highly desirable to unravel their detailed functioning. Very recently, the first multiple *operando* spectroscopic approach in the context of gas sensors was introduced combining resistance measurements with UV-Vis, Raman, and gas-phase infrared spectroscopy in one experimental setup [6]. This multiple spectroscopic approach allows monitoring the presence of adsorbates and hydroxy groups (Raman) and the number of oxygen vacancies (UV-Vis), while simultaneously capturing the gas-phase composition (IR). The potential of this new approach was demonstrated for SnO_2_, the most commonly used gas sensors material, in the context of EtOH detection [6]. This is illustrated in Figure 12, which shows the temporal evolution of spectroscopic data (Raman, UV-Vis, IR), and sensor resistance for varying gas atmospheres and temperatures. Raman spectroscopic experiments were performed using 514.5 nm excitation, and Raman band intensities were obtained by area integration, based on Raman spectra, recorded over 10 min (as indicated by the bars). UV-Vis spectra were taken within 1 min directly after the Raman spectra and are represented by the reflectance at 525 nm.

In the following, the correlation of the sensor resistance with the spectroscopic features will be briefly described, focusing on the Raman results. For details please refer to [6]. Exposing the gas sensor to a reducing gas, i.e., 250 ppm EtOH/N_2_ at 190 °C results in the expected decrease in resistance due to the release of electrons into the conduction band, while carbon dioxide, acetaldehyde, and water (not shown) are detected as reaction products. As discussed in Section 4.1, the formation of acetaldehyde has previously been proposed to proceed via adsorbed ethoxy undergoing dehydrogenation. Besides, the presence of EtOH/N_2_ leads to a strong reflectance decrease from 100% to 84% as a result of oxygen-vacancy formation, i.e., the reduction of SnO_2_ [47]. Whereas, Raman data reveals the presence of acetate species as a result of the reaction between (adsorbed) ethanol and surface hydroxy groups. When the feed is switched back to pure nitrogen, a small increase in the resistance and decrease in the acetate Raman band is observed. As discussed previously, this behavior originates from the formation of stable acetate adsorbates formed during EtOH/N_2_ exposure, preventing the sensor from returning to its initial state [5,34]. The addition of oxygen to the feed induces a resistance increase as a result of reoxidation of the surface, removing electrons from the conduction band. This is reflected by Raman spectra exhibiting a decrease in the acetate and an increase in the formate signal, thus, indicating the partial decomposition of acetate into formate, and by UV-Vis spectra showing an increase of the reflectance (to 88%). When switching to 250 ppm EtOH/air, the observed resistance and reflectance (to 87%) decrease is less pronounced than in EtOH/N_2_ due to permanent reoxidation by oxygen. The presence of oxygen results in a larger conversion of EtOH and the formation of larger amounts of carbon dioxide. Adsorbed species acetates are detected in the Raman spectra as in case of EtOH/N_2_ exposure. 

Prior to gas sensing experiments at 325 °C, SnO_2_ was heated to 400 °C to remove all adsorbates from the surface, as indicated by the sharp CO_2_ gas-phase signal and the return of the resistance to its initial value before EtOH exposure. Exposure to 250 ppm EtOH in air at 325 °C leads to a significantly higher CO_2_ concentration, as compared to 190 °C, while acetaldehyde shows a similar concentration. Under these conditions of higher EtOH conversion, no adsorbates are detected in the Raman spectra. In the presence of EtOH, both resistance and reflectance show a reversible decrease. Switching to 250 ppm EtOH/N_2_ at 325 °C induces a significantly stronger decrease in the resistance and the reflectance (from 91% to 67%) than in EtOH/air, as a consequence of the missing reoxidation of the SnO_2_ sensor by oxygen. The smaller availability of oxygen also implies a smaller ethanol conversion and a significantly larger fraction of acetaldehyde. New carbon-related Raman bands at around 1350 cm^−1^ (D) and 1575 cm^−1^ (G) are observed, indicating the decomposition of ethanol on the sensor surface, and preventing other hydroxy and/or adsorbate-related (v_C-H_) Raman bands from being detected. Upon switching back to pure nitrogen, the carbon-induced changes in the Raman spectra disappear, and the resistance increases accompanied by an increase of the reflectance to 84%. The observed changes indicate that at elevated temperatures of the gas sensor, bulk to surface diffusion of oxygen allows for the reoxidation of the sensor. When adding oxygen, the surface of the sensor is further reoxidized, leading to an increase of the resistance to the initial state in synthetic air, caused by the removal of electrons from the conduction band. The surface reoxidation is spectroscopically evidenced by the increase in reflectance to 90%.

**Figure 13:**
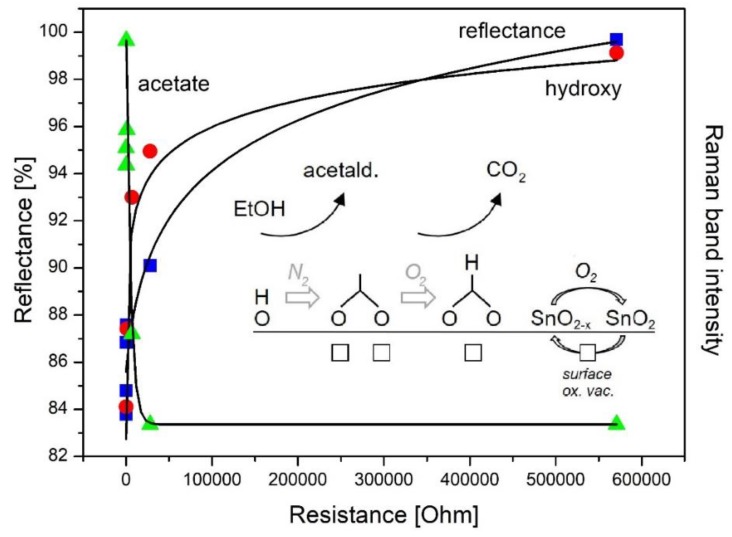
Proposed mechanism of the ethanol gas sensing of SnO_2_. Reflectance and Raman band intensities of hydroxy and acetate species as a function of sensor resistance, together with least-squares fits to the experimental data. Reproduced from [6].

Summarizing, our findings from multiple *operando* spectroscopy show that the sensor response is correlated with the number of surface oxygen vacancies and the nature of the adsorbates. In fact, as illustrated in Figure 13, the resistance decreases as the number of oxygen vacancies and the formation of adsorbed acetate increases, which in turn, is related to the consumption of hydroxy species. The direct correlation of the resistance with the concentration of oxygen vacancies can be further specified based on recent Raman studies, reporting that the subsurface oxygen vacancy dynamics is not relevant for gas sensing in CeO_2_ gas sensors (see Section 4.1) [36]. Thus, we propose that the resistance changes observed in SnO_2_ gas sensors originate from oxygen vacancies located at the surface of the sensor. 

## 5. Conclusions and Outlook

In this review we summarized the impact of vibrational Raman spectroscopy on deepening our mechanistic understanding of working metal-oxide gas sensors by starting from initial In situ applications to the current state of knowledge using multiple *operando* spectroscopic approaches. As discussed above, Raman spectroscopy can be applied to a wide range of sensor materials revealing valuable information on the (sub)surface structure, including hydroxyl groups and the presence of adsorbates. In favorable cases, the (oxidation) state and oxygen vacancies in the subsurface region of the sensor material, as well as the state of the metal dopand, are accessible. Thus, by vibrational Raman spectroscopy, the properties of the surface and the underlying solid can be probed by one experimental technique.

Besides the importance of oxygen vacancies (accessible by UV-Vis and in some cases also by Raman spectroscopy) for the gas sensing mechanism, the sensor response has been shown to depend on the presence of adsorbates (accessible by Raman and IR spectroscopy) highlighting the relevance of ionosorption in gas sensing. For example, for ethanol gas sensing, *operando* Raman results have shown a decrease in resistance for increasing acetate concentration, as discussed above in great detail.

We conclude this review with a brief outlook on potential future developments of vibrational Raman spectroscopy in the context of metal-oxide gas sensors. While, previous work has demonstrated the versatility of Raman spectroscopy in addressing important aspects of the gas sensor mechanism, its wider application is still hampered by the low spread of Raman spectrometers. However, modern Raman spectrometers are easy to use and can be readily applied much like IR spectrometers. 

So far Raman spectroscopic studies on gas sensors have been restricted to visible or NIR excitation. However, the use of UV excitation may offer several advantages as has been demonstrated in the context of catalytic materials [48,49]: First, fluorescence effects can be prevented. As for UV excitation, the Raman signal is widely separated in energy from the fluorescence emission. Second, resonance Raman effects may be exploited by the excitation of electronic transitions leading to an enhancement of the Raman signal. Third, in principle, the signal should increase due to the strong frequency dependence of the Raman intensity; however, self-absorption effects may become important. 

A further aspect to be considered in the context of future developments is the application of theoretical calculations to facilitate Raman band assignments. This has been recently demonstrated for oxygen (vacancy)-related features in ceria materials [36], as discussed above. Much more theoretical work will be necessary to fully explore the Raman features of common gas sensor materials, such as SnO_2_, WO_3_ or In_2_O_3_. The advantage of combining *operando* Raman experiments with theory is expected to elucidate new aspects of the gas-sensing mechanism in the future.

## Figures and Tables

**Table 1:** Overview of In situ and *operando* Raman studies on metal-oxide gas sensors.

	Metal Oxide	Target Gas
**In situ**	SnO_2_	H_2_S [22]
		NO_2_ [25,31]
	CuO/SnO_2_	H_2_S [23]
	S/SnO_2_	NO_2_ [32]
	WO_3_	CH_4_, CO, NO_2_ [24]
		H_2_ [29]
	ZnO	H_2_ [30]
	BaCeO_3_	Air, N_2_, CO_2_ [28]
	V_2_O_5_	NH_3_ [27]
	TiO_2_	NO_2_ [31]
***Operando***	SnO_2_	EtOH [6]
	In_2_O_3_	EtOH, acetald., ethene, CO [5,34,35]
	Ag/In_2_O_3_	EtOH, CO [35]
	CeO_2_	EtOH [36]
